# Biography of *Vitis* genomics: recent advances and prospective

**DOI:** 10.1093/hr/uhae128

**Published:** 2024-05-07

**Authors:** Yi Wang, Kangyi Ding, Huayang Li, Yangfu Kuang, Zhenchang Liang

**Affiliations:** State Key Laboratory of Plant Diversity and Specialty Crops and Beijing Key Laboratory of Grape Science and Enology, Institute of Botany, the Chinese Academy of Sciences, No.20 Nanxincun, Xiangshan, Haidian, Beijing 100093, China; China National Botanical Garden, Beijing 100093, China; State Key Laboratory of Plant Diversity and Specialty Crops and Beijing Key Laboratory of Grape Science and Enology, Institute of Botany, the Chinese Academy of Sciences, No.20 Nanxincun, Xiangshan, Haidian, Beijing 100093, China; China National Botanical Garden, Beijing 100093, China; University of Chinese Academy of Sciences, Beijing 100049, China; State Key Laboratory of Plant Diversity and Specialty Crops and Beijing Key Laboratory of Grape Science and Enology, Institute of Botany, the Chinese Academy of Sciences, No.20 Nanxincun, Xiangshan, Haidian, Beijing 100093, China; China National Botanical Garden, Beijing 100093, China; University of Chinese Academy of Sciences, Beijing 100049, China; State Key Laboratory of Plant Diversity and Specialty Crops and Beijing Key Laboratory of Grape Science and Enology, Institute of Botany, the Chinese Academy of Sciences, No.20 Nanxincun, Xiangshan, Haidian, Beijing 100093, China; China National Botanical Garden, Beijing 100093, China; State Key Laboratory of Plant Diversity and Specialty Crops and Beijing Key Laboratory of Grape Science and Enology, Institute of Botany, the Chinese Academy of Sciences, No.20 Nanxincun, Xiangshan, Haidian, Beijing 100093, China; China National Botanical Garden, Beijing 100093, China

## Abstract

The grape genome is the basis for grape studies and breeding, and is also important for grape industries. In the last two decades, more than 44 grape genomes have been sequenced. Based on these genomes, researchers have made substantial progress in understanding the mechanism of biotic and abiotic resistance, berry quality formation, and breeding strategies. In addition, this work has provided essential data for future pangenome analyses. Apart from *de novo* assembled genomes, more than six whole-genome sequencing projects have provided datasets comprising almost 5000 accessions. Based on these datasets, researchers have explored the domestication and origins of the grape and clarified the gene flow that occurred during its dispersed history. Moreover, genome-wide association studies and other methods have been used to identify more than 900 genes related to resistance, quality, and developmental phases of grape. These findings have benefited grape studies and provide some basis for smart genomic selection breeding. Moreover, the grape genome has played a great role in grape studies and the grape industry, and the importance of genomics will increase sharply in the future.

## Introduction

Grape is one of the most important horticultural crops in the world, with high economic and cultural value. It plays a critical role in the history and daily life of the world [1]. However, the grape industry has been greatly affected by the drastic changes of the climate in the last 20 years. Therefore, breeding high-quality environment-adaptive varieties and conducting basic studies on the quality and resistance of the berry have been the two main aspects of grape studies. Both aspects require grape genomes, so grape genomes could prove fundamental to breeding and basic studies, making them crucial to the grape industry.

## Grape genomics provide the basis of grape studies

The first grape genome was published in 2007, and two grape genome projects unveiled the genome sequences of *Vitis vinifera* cv. ‘Pinot Noir’ (PN40024 and ENTAV115) [2, 3]. Since then, grape studies entered the genome era, and PN40024 became the most popular reference genome in grape studies. In the last 16 years, the PN40024 genome has been updated at least four times, with more than nine annotated versions [4, 5] (Table 1). Finally, the high-quality telomere-to-telomere (T2T) genome PN40024 was released in 2023 [6]. This genome was nearly complete, with a genome size of 494.87 Mb and gene number of 37 543. This assembly also provides more lost information, which will greatly benefit grape studies compared with the previous version. But recent studies based on the genome datasets have suggested that PN40024 was not the inbred offspring of ‘Pinot Noir’ but resulted from nine selfings of ‘Helfensteiner’ [5].

**Table 1:** Assembled and annotated versions of PN40024 genomes

**Year**	**Genome version**	**Genome size (Mb)**	**Number of genes**	**Annotation version**	**Sample**	**References**
2007	8X	497.51	30 434	8X	GSVIVT00000001001	[2]
2009	12X.v0	486.20	31 845	V0	GSVIVT01000001001	[4]
2009	12X.v0	486.20	31 845	V1,V1R	VIT_10s0116g00060.t01	[4]
2009	12X.v0	486.20	31 845	V2,V2.1	VIT_201s0011g00040.1	[4]
2017	12X.v2	486.20	42 413	V3	Vitvi09g02070	[81]
2020	PN40024.v4	475.60	35 230	V4	Vitvi01g04000	[5]
2023	PN_T2T	494.87	37 534	PN_T2T	Vitis12g01659	[6]

**Figure 1:**
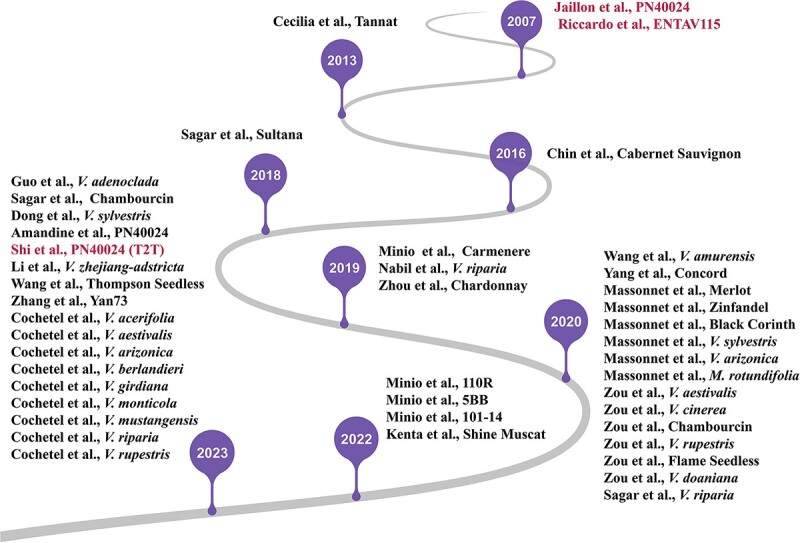
The history of grape genome sequencing.

With the development of sequencing technology, at least 44 grape genomes have been released so far, including at least 16 for *V. vinifera* subsp. *vinifera*, 21 for wild *Vitis* species, and 9 for interspecific hybrids [2, 7–21] (Fig. 1). Studies on these genomes focused on grape berry quality (sugar, acid, aroma, pigment, and so on) [7, 12, 18], biotic and abiotic resistance [2, 13], and future breeding strategies [9, 22]. Through these genomes, researchers explored the mechanisms of disease resistance, high tannin content, and new breeding strategies. 

Among these sequenced genomes, that of *V. vinifera* has attracted considerable attention because of the widespread cultivation of this grape, including the first sequenced genome, PN40024 [2]. The sequence of PN40024 provides insight into the whole-genome triplication that occurred ~120–150 Mya. In this genome sequence, researchers also found many nucleotide-binding site (*NBS*) genes that were related to disease resistance. The genome of ‘Thompson Seedless’ reveals a decrease in TIR-NB-LRR (TNL) and CC-NB-LRR (CNL) family genes, which potentially impacts disease susceptibility, while transcriptome analysis underscores the pivotal role of nucleotide-binding leucine-rich repeat (*NLR*) genes in grapevine defense against powdery mildew [21]. Apart from evolution and disease resistance, berry and wine quality are always the first concern of grape studies. During the study of the ‘Tannat’ genome, which contained more polyphenolic compounds than other cultivars [7], researchers identified 1873 specific genes in this new assembly and confirmed that these specific genes contributed up to 81.24% expression of enzymes involved in the synthesis of phenolic and polyphenolic compounds. After comparing the structural variations in ‘Chardonnay’, researchers explained the mechanism by which the structural variation contributes to the diversity of berry color in grape [12]. The genome sequence of red-fleshed ‘Yan73’ also brought new insights into anthocyanin biosynthesis in the grape berry, helping our understanding of the coloring mechanism of teinturier grapes and providing guidance in breeding for high anthocyanin content [20].

Wild grape, which has high genetic diversity, has also been an important subject of grape genomic studies [10, 13, 18]. Wild *Vitis* species are widely found in the north of temperate zones and subtropics. These species contain high genetic diversity and some unique genes that are non-existent in modern cultivars. Therefore, the exploration of key genes only existing in these wild species has also been a main task of grape research [9, 10, 13, 18, 23]. In 2020, the chromosome-level genome of *V. amurensis*, which is known as the most cold-resistant among *Vitis* species, was released. Based on this genome, researchers have explored different response mechanisms for the chilling and freezing tolerance of *V. amurensis* [13]. Genome-wide association studies (GWAS) and RNA-seq analysis indicated that high efficiency of sugar metabolism in dormant buds contributes to high cold tolerance during freezing winters [13]. Apart from cold tolerance, sex determination is also important in grape breeding. Researchers have compared the genome of *V. vinifera* ssp. *sylvestris* and other cultivars and enhanced the understanding of the genetic basis of sex determination in grape [9]. Additionally, some special *Vitis* species, such as *V. adenoclada* and *V. riparia*, and some other wild species have also been sequenced. The study of these wild accessions has mainly focused on the high resistance, sex domestication, and metabolism of phenols [9, 16–18]. In addition, some special grapes, which were considered endangered worldwide, have attracted the interest of researchers. The genome sequencing of *V. zhejiang-adstricta* not only safeguarded biodiversity, but also enriched the conservation and utilization of wild grape resources. Meanwhile, genome studies have identified the slow growth and endangered characteristics of species by analyzing the genes gained and lost during evolution and clarified the characteristics of disease resistance obtained through cumulative variation. Besides, a reference-unbiased super-pangenome work on nine assembled genomes of wild North American grapes has revealed both inter- and intraspecific genomic variation, along with accurately estimating similarity between hybrids and their parent species. Subsequent pan-GWAS successfully pinpointed loci linked to salt tolerance, showcasing the potential for implementing this reference-unbiased super-pangenome to expedite crop breeding efforts [19].

Collectively, wild grape species serve as a perfect gene pool, especially for biotic and abiotic resistance. Their genomes could provide new insights into environmental adaptability and cultivar improvement of grapes. In enhancing the adaptability of modern grape varieties, researchers aim to explore useful genes from wild species genomes and introduce key genes into cultivars through hybrid breeding or other methods.

## Re-sequencing datasets elaborated the domestication history and provided guidance for modern breeding methods

Compared with *de novo* assembled genomes, whole-genome sequencing (WGS) could find genetic variations such as SNPs and InDels at low cost. Variations identified based on high-throughput sequencing could help researchers understand the diversity of populations and explore their mysteries. In grape, several WGS projects performed in the last 6 years focused on the origin and domestication history of cultivated grape [23–26], the effect of clonal propagation on grape breeding and evolution [27–29], gene flow and environmental adaption [23, 25, 29, 30], and gene identification of genes for traits characteristic of grape [13, 17, 24, 31].

It was thought that cultivated grape originated or was domesticated in the Pan-Black Sea region, and studies based on 472 grape accessions confirmed this opinion [24]. However, another study based on 204 *V. vinifera* accessions revealed that the modern cultivated grape was initially domesticated in East Asia, and then spread to Europe and other regions [32]. Both results were reliable, which led to new doubts about the origin of cultivated grape. Considering that both projects were limited by the population size, a huge grape genome re-sequencing project, which contained 3525 accessions of *V. vinifera* and its wild relatives, provides new insight into the domestication history of grape [23]. Grape was believed to be domesticated 11 000 years ago in Western Asia and the Caucasus to yield table and wine grapes through two independent domestication events, and this result was confirmed by other genomic evidence [29]. However, recent studies have also found evidence for a single domestication of grapevine with introgression from the wild relative to wine grapes, but not to table grapes. So, the origin of grape domestication is still highly controversial [33]. All these projects aimed to systematically elaborate the domestication history of grape step by step and have provided new insight into crop domestication [23], but how to achieve this goal still needs more data and a lot of work.

Based on the huge number of variations, researchers also aim to explore what happened during the long history of breeding and what should be done in the future. Based on the re-sequencing data of 28 archaeological grape seeds, researchers found that grape has had at least 900 years of uninterrupted vegetative propagation [28]. In addition, the variation of 28 accessions confirmed that clonal propagation leads to the accumulation of recessive deleterious mutations but without decreasing fitness [27]. Although clonal propagation has a long history, researchers have still confirmed that gene flow from Iberia or American wild species contributes to the genetic diversity and environmental adaption of the modern cultivars [23, 25, 26, 29]. Moreover, a recent study based on 345 accessions has confirmed a significant signal of gene flow between wild species and cultivars, highlighting that hybrid breeding, especially hybrids with wild species, is essential for grapes [29]. All these works indicated that the long history of clonal propagation in grape breeding is disadvantageous, and hybridization with wild species is a potential method to improve grape breeding in the future.

## Whole-genome sequencing of germplasms shows great application potential in gene identification

In many WGS studies, high-density variation maps were also used to process GWAS or a high-density linkage map based on QTL mapping. These methods have been primarily used to identify key genes of a particular phenotypic trait in recent studies. In grapes, a number of gene identification studies based on sequencing have also been performed, including Restriction-site Associated DNA Sequence (RAD-seq), Genotyping-by-Sequencing (GBS), high-throughput microarray, Bulked Segregant Analysis (BSA), and re-sequencing [24, 31, 32, 34–36, 80]. Based on these datasets, the forward genetics method has been used to identify key regulated genes in grape studies, particularly in berry quality [24, 31, 34], content of secondary metabolites [24, 35], and biotic and abiotic resistance [13, 36].

In the last decade, researchers have widely used high-density linkage maps to explore the regulation mechanisms of seed development, primary or secondary metabolites, and disease resistance. In exploring the mechanism of development of seedless table grapes, researchers have constructed a genetic map using SSR and SNP markers and confirmed that *VvAGL11* was associated with the origin of seedless grapes [37]. Using 1254 SNP markers obtained from the GBS datasets, Chen *et al*. constructed a high-density genetic map and identified 134 genes related to the content of sugar and acid in grapes [34]. Based on a 3332-SNP linkage map, Zhang *et al*. found that *VvbZIP61* could contribute to the content of monoterpenes in grape berries [35]. Using a linkage map of *V. vinifera* and *V. davidii*, researchers found that *PR1* could enhance the tolerance of white rot [38].

Apart from map-based QTL identification, a number of GWAS analyses have also been conducted to identify key genes for some important traits. In 2019, based on the genetic map of 472 accessions, GWAS analysis was performed on 24 grapevine phenotypes, and many genes related to the quality traits of grapes were found [24]. Berry traits also draw the attention of researchers. Using 32 311 variations from 179 grape accessions, researchers identified several key genes related to berry-related traits [31]. A recent study utilizing 167 accessions of *Vitis arizonica* has identified key resistance genes to *Xylella fastidiosa*. Apart from WGS, some new sequencing technologies were also used to identify genes, such as RNase H2 enzyme-dependent amplicon sequencing (rhAmpSeq), which has been used to explore 52 QTLs that are related to 12 berry traits [17].

**Figure 2:**
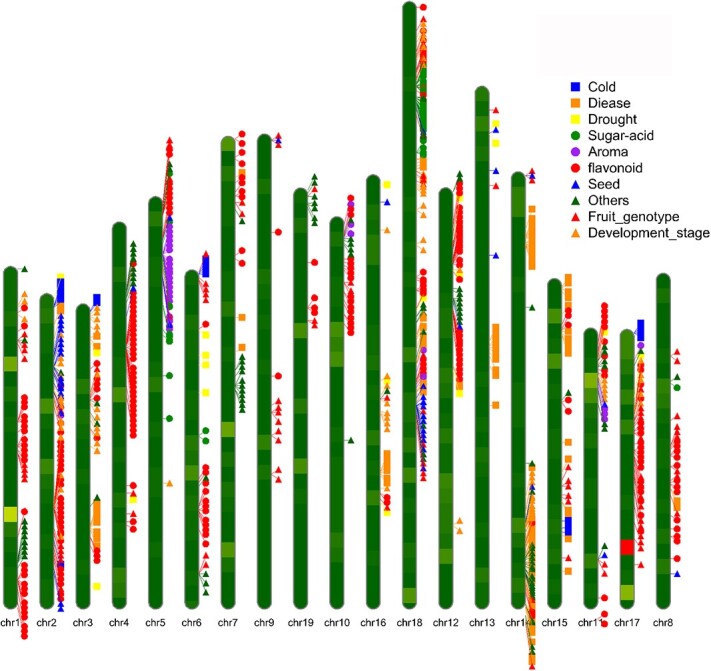
Distribution of candidate functional genes of diverse agronomic phenotypes.

In the past two decades, a number of studies on grape gene identification have been performed and 941 genes (Fig. 2, Supplementary Data Table S1) have been identified, which were related to all traits of grape, including berry development [39–45], berry quality regulation [31, 37, 42, 46–59], fruit genotypes [60], sugar and acid content [34, 52, 61], aroma and flavonoids [48, 62–69], and biotic and abiotic resistance [70–77]. Among these genes, the biosynthesis of anthocyanins and flavonoids has received considerable attention from researchers, with 277 candidate genes having been identified [62–69], followed by disease resistance (123 genes) [72–76], and plant and berry development (83 genes) [31, 39–45]. All these genes were important genetic resources for grape breeding, and these studies provide a solid foundation for grape genome-selective breeding.

## Utilization and future of grape genomics

In the post-genomics era of grape, new strategies should be provided to cope with the challenge caused by the enormous datasets and emerging new technologies. Thus, comprehensive databases or platforms that could provide efficient and visual representation information to researchers are necessary. To date, several grape databases have been published, such as VitisGDB [78], Grape-RNA [79], and Grapedia. However, a more comprehensive and more humanized database is still necessary to hold and exhibit the grape genome and related datasets, especially for researchers without experience in big data analysis. In addition, the deep mining of these datasets is a big problem for researchers; therefore, comprehensive databases could help people to browse the data conveniently and utilize these data.

Grape genomics projects have primarily aimed to provide guidance for grape improvement breeding and related research. Genomic selection or marker-assisted breeding is the primary way to achieve this goal. Therefore, deep mining the WGS data of germplasms and identifying genes related to quality and resistance phenotypes could help people achieve this goal. Considering the complexity of quantitative traits, a number of markers should be considered. Furthermore, a new method that could detect the amount of markers simultaneously at relatively low cost is necessary, and the low-density array is an excellent choice.

With the development of sequencing technologies, the cost of high-quality T2T genomes is sharply decreasing, and the assembled genome could provide more information, such as chromosome-level structure variation and long terminal repeats. In addition, WGS and assembly could provide almost all information about individuals, detecting more variation than previous methods. In grapes, the main cultivars have relatively low diversity, and some varieties have a number of lines that contain few variations. Therefore, pangenome analysis of these genomes could find rare variation and provide new insights into grape breeding and more information for modern improvement breeding.

In brief, grape genomics has greatly benefitted grape breeding and research. However, grape genomics is still being rapidly developed, and it primarily aims to identify key genes and loci related to traits and apply them to breeding. In the post-genomic era, the utilization of grape genomic datasets and development of new tools to make analysis more accurate and more convenient are a challenge as well as a big chance for grape breeding.

## Supplementary Material

Web_Material_uhae128
